# Burden and patterns of dyslipidaemia among adult Ghanaians: A systematic review

**DOI:** 10.1371/journal.pone.0350185

**Published:** 2026-05-28

**Authors:** Richmond Owusu Ateko, Andrew Decker, Afua Bontu Adjei, Samuel Mawuli Adadey, Eric Nana Yaw Nyarko, Nicholas Ekow Thomford

**Affiliations:** 1 Department of Chemical Pathology, University of Ghana Medical School, University of Ghana, Accra, Ghana; 2 Franklyn Medical Centre, Accra, Ghana; 3 Department of Biochemistry and Molecular Medicine, School of Medicine, University for Development Studies, Tamale, Ghana; 4 Division of Chemical Pathology, Department of Pathology, Faculty of Health Sciences, University of Cape Town, Cape Town, South Africa; 5 Pharmacogenomics and Genomic Medicine Group and Laboratory, School of Medical Sciences, College of Health and Allied Sciences, University of Cape Coast, Cape Coast, Ghana; 6 Department of Medical Biochemistry, School of Medical Sciences, College of Health and Allied Sciences, University of Cape Coast, Cape Coast, Ghana; 7 Medical and Scientific Research Centre, University of Ghana Medical Centre, Accra, Ghana; Tehran University of Medical Sciences, IRAN, ISLAMIC REPUBLIC OF

## Abstract

**Background:**

Dyslipidaemia is a major modifiable risk factor for cardiovascular disease and has become an increasing public health concern in sub-Saharan Africa. Rapid urbanisation, dietary transitions, and sedentary lifestyles have contributed to the rising incidence of cardiometabolic diseases in Ghana. However, evidence on the overall burden and patterns of dyslipidaemia in adults remains unclear. This systematic review aimed to synthesise the evidence on the prevalence and lipid profile patterns of adult Ghanaians.

**Methods:**

A comprehensive search of PubMed, Scopus, Web of Science, African Journals Online, Africa-Wide Information and African Index Medicus databases was conducted for studies published between January 1980 and November 2025. Eligible studies reported the prevalence or distribution of dyslipidaemia in Ghanaian adults (≥ 18 years). Two reviewers independently screened, extracted, and assessed the quality of included studies. Findings were synthesised narratively and summarised in descriptive tables and figures, due to heterogeneity in study designs and data reporting.

**Results:**

Twenty-four studies published between 2003 and 2023, comprising approximately 11,400 participants, met the inclusion criteria. Across the included studies, the reported prevalence of dyslipidaemia ranged from 3.0% to 72.4%, reflecting differences in study populations, diagnostic criteria and study settings. Low high-density lipoprotein cholesterol (HDL-C) was the most frequent abnormality, followed by elevated total and low-density lipoprotein cholesterol (LDL-C), whereas hypertriglyceridaemia was the least common. Studies from southern and middle regions—particularly Ashanti, Brong-Ahafo, and Greater Accra—reported a higher dyslipidaemia prevalence than those from northern Ghana. Considerable variation in diagnostic criteria, study populations and sampling strategies limited comparability and precluded meta-analysis.

**Conclusion:**

Dyslipidaemia is common among adult Ghanaians, with low HDL-C emerging as the predominant abnormality. Methodological differences and sampling biases limit the precise estimation of the national burden, but the available evidence indicates a growing cardiometabolic risk. Future studies should prioritise community-based sampling and context-appropriate lipid thresholds to support evidence-based cardiovascular risk-reduction strategies.

## Introduction

Dyslipidaemia, a major modifiable risk factor for cardiovascular diseases (CVD), is characterised by abnormal levels of lipids and lipoproteins in the blood, including elevated total cholesterol (TC), low-density lipoprotein cholesterol (LDL-C), triglycerides (TGs), and/or reduced high-density lipoprotein cholesterol (HDL-C) [1]. CVD remains the leading cause of morbidity and mortality globally, accounting for an increasing proportion of deaths in low- and middle-income countries (LMICs) [2,3].

In sub-Saharan Africa (SSA), the burden of dyslipidaemia has risen substantially in recent times due to rapid urbanisation, dietary transitions, and increasingly sedentary lifestyles [4,5]. A study by Noubiap et al. reported continental prevalence estimates of 25% for elevated TC, 37% for low HDL-C, 28.6% for elevated LDL-C, and 17% for elevated TG, with considerable inter-country variation [6]. Despite these figures, national-level data for several African countries, such as Ghana, remain limited or fragmented.

Ghana, like several countries in the region, is becoming a population in health transition, experiencing a significant epidemiological shift marked by a growing burden of non-communicable diseases (NCDs), including cardiovascular disorders [7]. Lifestyle and nutritional changes associated with urbanisation have contributed to the increased prevalence of cardiometabolic risk factors, including obesity, hypertension, diabetes, and dyslipidaemia [5,8,9]. Several studies conducted across different populations have reported varying dyslipidaemia frequencies, reflecting differences in study design, diagnostic criteria, and population characteristics. However, despite these individual studies, a consolidated national synthesis of dyslipidaemia prevalence in Ghana is still lacking.

Therefore, this systematic review aimed to synthesise the available evidence on the burden and patterns of dyslipidaemia among adult Ghanaians, focusing on the reported prevalence, lipid profile subtypes, and variations in demographic and clinical factors. By collating and analysing existing studies, this review seeks to provide a clearer picture of the national situation and identify critical knowledge gaps that can inform policy decisions, health interventions, and future research on cardiovascular disease prevention in Ghana.

## Materials and methods

### Protocol and registration

This review followed the Preferred Reporting Items for Systematic Reviews and Meta-Analyses (PRISMA 2020) guidelines [10]. The protocol was registered in the International Prospective Register of Systematic Reviews (PROSPERO, registration number: CRD42020198175).

### Search strategy

A comprehensive literature search was conducted to identify studies that reported on the prevalence or distribution of dyslipidaemia among adult Ghanaians. The databases searched included PubMed (MEDLINE), Scopus, Web of Science, African Wide Information, African Journals Online, and African Index Medicus. The search covered the period from January 1980 to July 2025. The search was updated in November 2025.

The search terms combined controlled vocabulary (MeSH) and keywords related to dyslipidaemia and Ghana. The core search string for PubMed is as follows:

(“Dyslipidemias” [Mesh] OR dyslipidemia* OR dyslipidaemia* OR hyperlipidemia* OR hyperlipidaemia* OR “lipid disorder*” OR hypercholesterolemia* OR hypercholesterolaemia* OR hypertriglyceridemia* OR hypertriglyceridaemia* OR “low HDL” OR “high LDL” OR “abnormal lipid*”) AND (“Prevalence” [Mesh] OR prevalence OR epidemiology OR frequency OR magnitude OR burden OR “cross-sectional” OR “population-based”) AND (“Ghana” [Mesh] OR Ghana* OR “Ghanaian adult*” OR “adult population in Ghana”).

Search strings were adapted for Scopus, Web of Science, African Journals Online, African Index Medicus, and Africa-Wide Information using the corresponding database syntax.

### Eligibility criteria

#### Inclusion criteria.

Studies were included if they were conducted in Ghana and involved adult participants aged 18 years or older. Only observational studies, including cross-sectional, cohort, and case-control designs, were included because they are the most appropriate for estimating disease prevalence. Eligible studies were required to report the prevalence or distribution of dyslipidaemia or abnormalities in any lipid fraction, namely TC, LDL-C, HDL-C, or TGs. To ensure methodological rigour, the studies also had to provide sufficient detail on sampling procedures and diagnostic criteria to assess quality and comparability.

#### Exclusion criteria.

Studies were excluded if they were conducted among Ghanaians residing outside the country or were based on an intervention or clinical trial design. Experimental studies or clinical trials were excluded as they are primarily designed to evaluate interventions rather than estimate population prevalence. Likewise, case series with fewer than 50 participants, qualitative studies, reviews, editorials, commentaries, and conference abstracts without accessible full texts were excluded from the study. Publications that lacked primary data, used unclear definitions of dyslipidaemia, or did not specify lipid measurement criteria were omitted from the final synthesis.

### Study selection

All records retrieved from the database searches were imported into Covidence (Veritas Health Innovation, Melbourne, Australia) for systematic review management. Duplicate records were automatically identified and removed from the platform before screening. Two reviewers (AD and ABA**)** independently screened the titles and abstracts of all the retrieved articles to identify studies that met the inclusion criteria. Full-text versions of the eligible papers were then retrieved and assessed in detail by two additional reviewers (SMA and ENYN) against the predefined inclusion and exclusion criteria. Any disagreements were resolved through discussion, and unresolved conflicts were adjudicated by a third reviewer (ROA).

A total of 247 records were identified through database searches, and no additional records were obtained from other sources. After removing 145 duplicates, 102 records were retained for further screening. After title and abstract review, 40 irrelevant records were excluded. The full texts of the 62 articles were retrieved and assessed for eligibility. Ultimately, 24 studies fulfilled all inclusion criteria and were included in the final synthesis.

**Fig 1:**
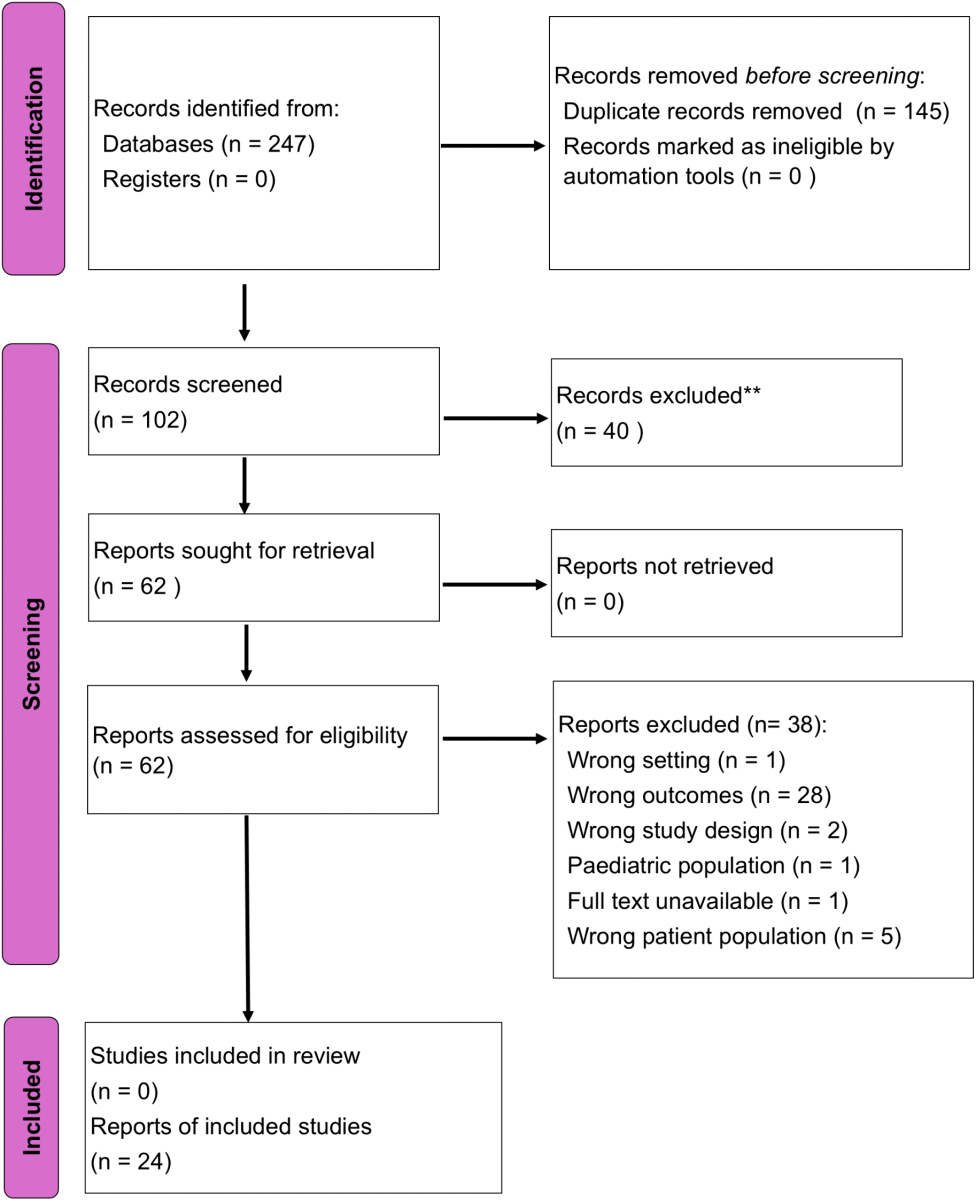
PRISMA flow diagram of the study selection process. The diagram summarises the number of studies identified, screened, excluded, and included in accordance with PRISMA 2020 guidelines.

The selection process is shown in Fig 1**.**

### Data extraction and synthesis

Data extraction was performed independently by two reviewers (ROA and SMA) using a standardised extraction form developed in Covidence (Veritas Health Innovation, Melbourne, Australia)**.** The extraction template was pilot-tested on a subset of studies to ensure its consistency and comprehensiveness. The following information was recorded for each eligible study.

- bibliographic details (first author, publication year, and study region),
- study design and setting (community- or hospital-based; urban, rural, or mixed),
- sample size and characteristics of participants (age range or mean age, and proportion of males),
- presence of comorbidities (such as hypertension, diabetes, or obesity),
- lipid parameters measured (total cholesterol, LDL-C, HDL-C, triglycerides),
- diagnostic criteria or cut-off values used to define dyslipidaemia, and
- reported the prevalence of overall and subtype-specific dyslipidaemia.

Any discrepancies between the reviewers during data extraction were discussed and resolved by consensus, with input from a third reviewer **(**ENYN**)** when required. The reviewers referred to the supplementary material when the data were incomplete or unclear. The extracted data were exported to Microsoft Excel 365 for descriptive synthesis and tabulation.

Data from the included studies were extracted and organised into a summary table based on the key study characteristics and lipid-related outcomes. The extracted variables included publication year, study design, population type, geographical region, sample size, diagnostic criteria, and reported lipid parameters (TC, LDL-C, HDL-C, and TG). The studies were subsequently grouped by participant category and geographical location, allowing for descriptive comparison of prevalence patterns. Substantial differences in study design, populations, diagnostic criteria, and reporting formats prevented meaningful statistical pooling; therefore, the studies were not combined quantitatively, and a narrative approach was used.

### Quality and risk of bias assessment

The methodological quality and risk of bias of all included studies were assessed using a modified version of the Newcastle-Ottawa Scale (NOS) [11] manual tool for non-randomised studies in meta-analyses. A structured set of questions from the risk of bias tool was applied consistently across all the studies. Each publication was rated on the quality and reporting standards of the biological and biomedical data presented (S3 Table).

## Results

### Study characteristics

**Fig 2:**
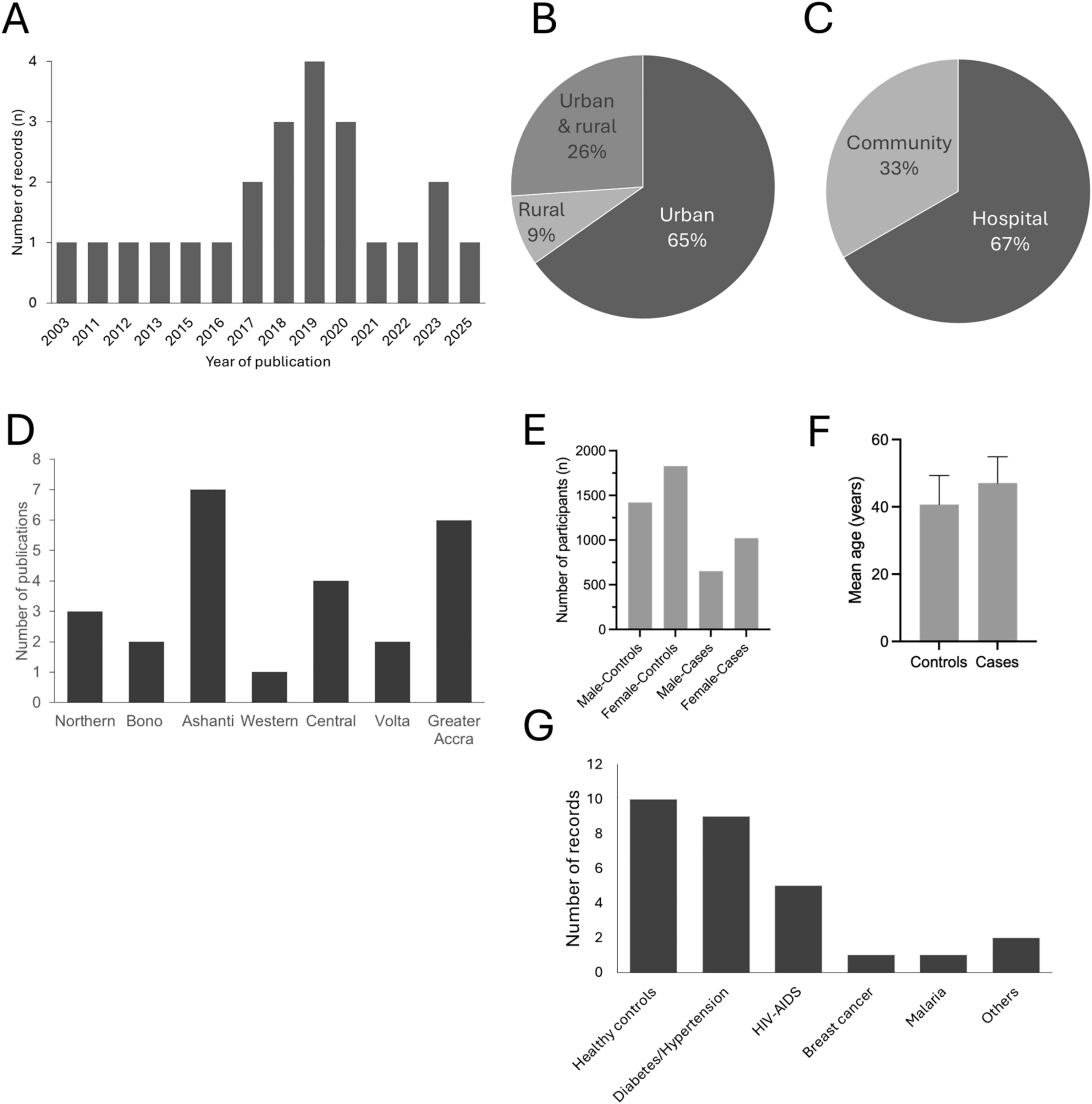
Characteristics of studies included in the systematic review. Panel A shows the publication year of the included studies. Panels B and C illustrate the study locations and settings. Panel D depicts the number of studies per region. Panels E and F show the sex and age distributions of study participants, and Panel G summarises the population categories recruited across studies (Numbers represent absolute counts).

A total of 24 studies published between 2003 and 2025 met the inclusion criteria and were included in this review. The characteristics of the included studies are summarised in Fig 1 and S2 Table, which outline the study locations, populations examined, and lipid parameters measured. Most studies were conducted in urban hospital settings**,** whereas a few included community-based or mixed (urban and rural) populations (Fig 2B and 2C). These studies represented all major geographical regions of Ghana, with the Ashanti, Greater Accra, and Central Regions being the most frequently studied (Fig 2D).

The sample size ranged from 70 to 3,317 participants**,** and the mean or median age ranged from 20 to 70 years. More females than males were recruited in all studies (Fig 2E). Both apparently healthy individuals and patient populations were represented, with no significant difference in the mean age (Fig 2F). A higher proportion of the studies focused on healthy controls**,** while others investigated patients with diabetes mellitus, HIV/AIDS**,** individuals with hypertension, or mixed clinical populations (Fig 2G). Across the included studies, 11.7% of the total study population were diabetics, while 5.8% had HIV/AIDS, and 1.0% were reported to have both HIV/AIDS and dyslipidaemia. Regarding the diagnostic criteria for dyslipidaemia, approximately two-thirds of the studies used the NCEP ATP III criteria. In contrast, the remaining studies defined dyslipidaemia according to ESC, ADA, or local institutional guidelines.

### Anthropometric characteristics of study participants

**Fig 3:**
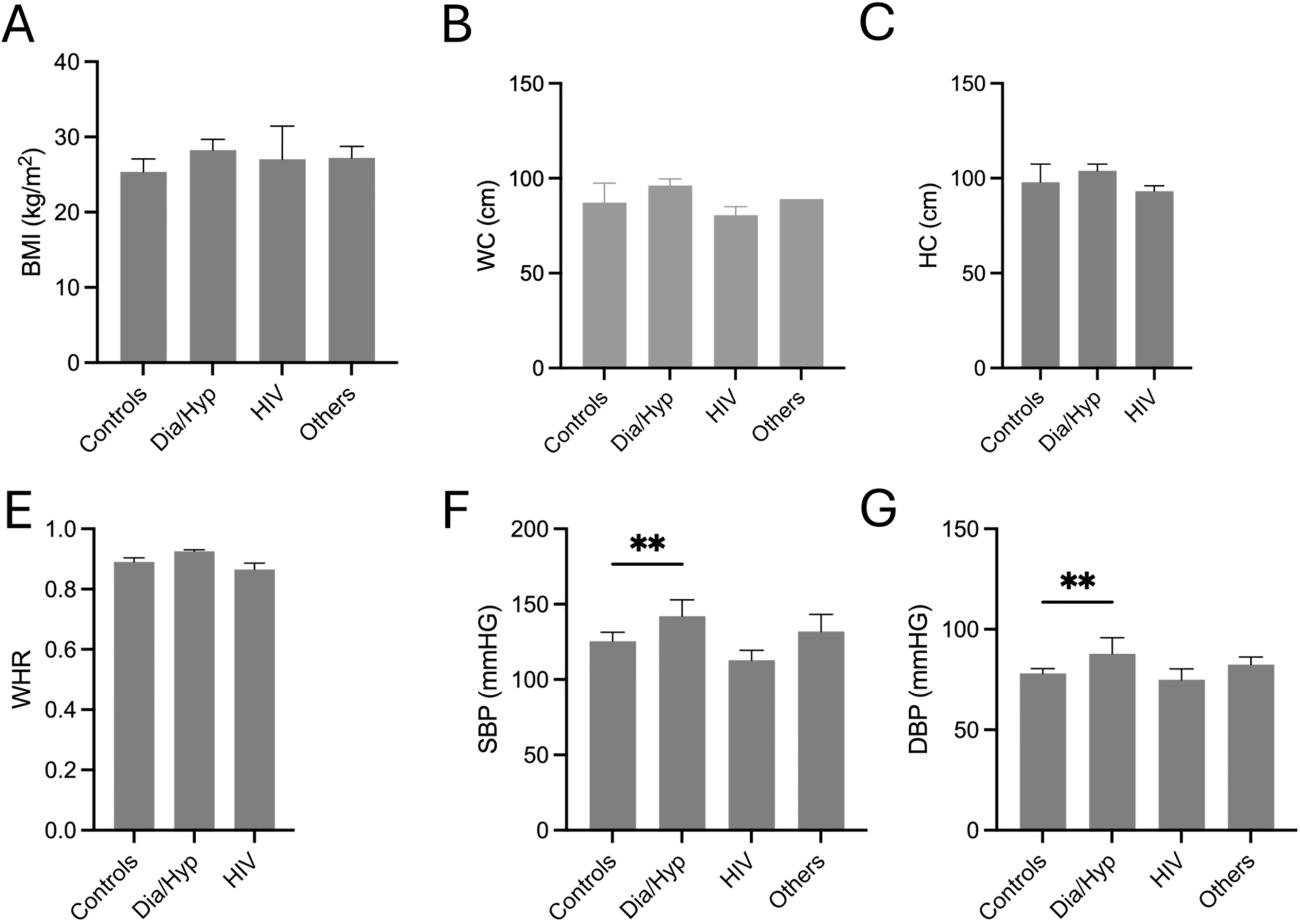
Anthropometric characteristics of study participants. The figure presents anthropometric variables retrieved from the included records: **(A)** BMI; (B) waist circumference; (C) hip circumference; (E) waist-to-hip ratio; (F) systolic blood pressure; and (G) diastolic blood pressure.

To examine the distributions of anthropometric variables, participants were grouped by diagnosis. ANOVA was used to examine differences between the patient and control groups. The analysis showed that the patients had BMI, waist circumference, and hip circumference values similar to those of controls (Fig 3). Subsequently, no significant difference in waist-to-hip circumference ratio was observed between the groups. However, the diabetes and hypertension groups had significantly higher systolic and diastolic blood pressure readings than the control group (Figs 3F and 3G).

### Lipid profile of study participants

Most studies (approximately two-thirds) used the National Cholesterol Education Program Adult Treatment Panel III (NCEP ATP III) criteria to define dyslipidaemia, whereas a few applied guidelines from the European Society of Cardiology (ESC)**,** the American Diabetes Association (ADA)**,** or local diagnostic standards such as those used at the Korle-Bu Teaching Hospital (KBTH)**.** Nearly all studies reported data on TC**,** TG**,** LDL-C**,** and HDL-C**,** although some failed to specify the cut-off thresholds (S2 Table). Overall, the included studies demonstrated substantial heterogeneity in study populations, diagnostic criteria, and settings**,** reflecting the diversity of dyslipidaemia research in Ghana.

**Fig 4:**
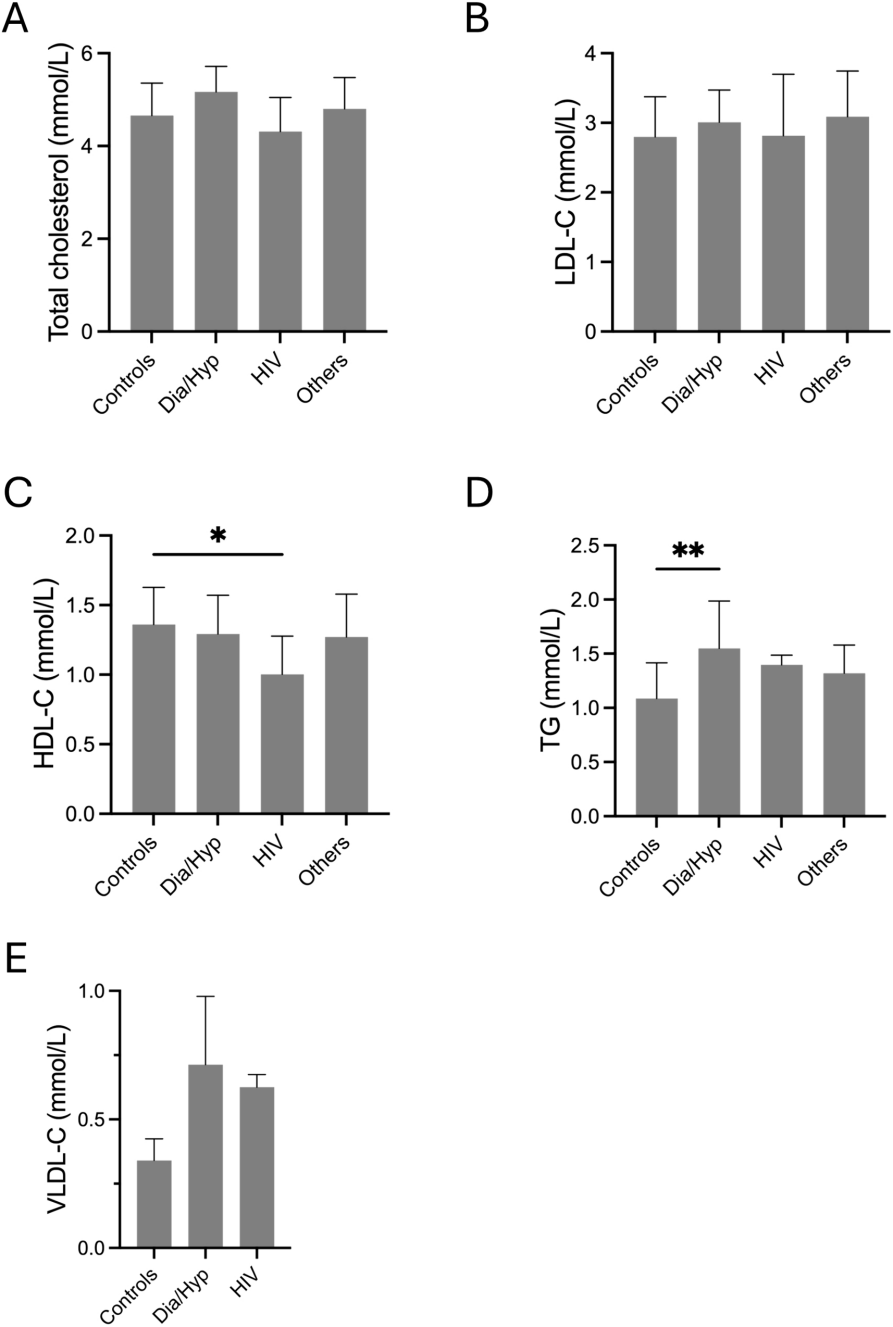
Lipid profiles of the participants. Panels A–E illustrate the distribution of lipid parameters across participant groups. Total cholesterol **(A)**, LDL-cholesterol **(B)**, HDL-cholesterol **(C)**, triglycerides **(D)**, and VLDL-cholesterol **(E)** were compared between controls and clinical groups, including diabetes/hypertension and HIV.

Lipid profile analysis revealed no significant differences in total cholesterol or LDL-C levels between the control and patient groups (Fig 4A, B). However, the HDL-C and TG levels were significantly different between the diabetic and control groups (Fig 4C, D). Although the VLDL-C levels were higher in the diabetes/hypertension and HIV groups than in the control group, these differences were not statistically significant (Fig 4E). The proportions of individuals with high TC, LDL-C, TG, and low HDL-C levels (based on retrieved records) were evaluated to compare the participant groups. Similarly, the frequency of low HDL-C levels was also assessed in these groups. Of all the variables examined, only high TC levels showed a statistically significant difference among the groups (p = 0.021) (Table 1).

**Table 1:** Distribution of Lipid Profile Abnormalities Across Participant Groups.

	Number of participants (%)		ANOVA	
	Group	Mean (range)	F-statistic	p-value
High TC	Controls	17.90 (4.02-36.50)	3.866	0.0202
	Diabetics/Hypertension	32.08 (32.41-62.79)		
	HIV	9.04 (0.33-38.98)		
	Others	29.55 (1.86-59.67)		
High LDL-C	Controls	26.27 (3.85-67.50)	0.9207	0.4446
	Diabetics/Hypertension	23.45 (7.44-47.44)		
	HIV	13.23 (0.33-3898)		
	Others	24.91 (2.48-61.08)		
Low HDL-C	Controls	26.42 (3.57-60.30)	0.5069	0.6807
	Diabetics/Hypertension	23.66 (2.83-66.51)		
	HIV	28.02 (0.62-52.54)		
	Others	14.98 (7.45-24.04)		
High TG	Controls	7.52 (1.92-15.63)	1.293	0.2964
	Diabetics/Hypertension	13.25 (3.20-48.37)		
	HIV	8.39 (2.48-13.89)		
	Others	15.42 (8.07-32.08)		

Values are presented as mean prevalence (%) with range across studies in parentheses. TC: Total cholesterol; LDL**-**C: Low-density lipoprotein cholesterol; HDL-C: High-density lipoprotein cholesterol; TG: Triglycerides. p < 0.05 was considered statistically significant.

### Prevalence of dyslipidaemia

The reported prevalence of dyslipidaemia varies substantially across studies and population groups. Among apparently healthy adults, the overall prevalence ranged from 4.0% to 62.8%**,** whereas studies involving individuals with chronic conditions, such as diabetes mellitus or HIV infection, tended to show higher rates. Across most studies, low HDL-C was the most frequently reported abnormality, followed by elevated TC and LDL-C, whereas hypertriglyceridaemia was the least common lipid disturbance.

The prevalence of individual lipid abnormalities reported in the included studies is summarised in Table 2. Considerable variation was observed across the regions and study populations. Elevated total cholesterol ranged from approximately 2.3% to 62.8%, while elevated cholesterol ranged from 2.3% to 72.4%. Hypertriglyceridaemia generally showed lower prevalence across studies, ranging from about 2.1% to 48.4%. In contrast, HDL-C emerged as one of the most frequently reported lipid abnormalities, with prevalence estimates ranging from 7.6% to 91.5%. These findings highlight substantial heterogeneity in lipid abnormality patterns across the included studies.

**Table 2:** Regional distribution of dyslipidaemia and major lipid fractions reported in the included studies.

	Prevalence (%)				
Region	High TC	High TG	High LDL-C	Low HDL-C	Reference
Northern	4.02%	2.12%	5.55%	60.30%	Agongo 2018
	47.46%	24.58%	52.54%	91.50%	Bani 2020
Bono	62.80%	48.40%	47.40%	66.50%	Anto 2019
	21.30%	24.00%	22.17%	23.34%	Kodaman 2016
Ashanti	41.40%	5.30%	15.10%	21.10%	Asamoah-Boakye 2017
	45.00%	25.70%	72.40%	30.50%	Eghan 2003
	59.70%	32.10%	61.20%	16.50%	Micah 2012
	2.30%	20.30%	2.30%	50.82%	Ngala 2013
	34.40%	15.60%	50.40%	59.80%	Obirikorang 2015
	45.66%	11.60%	52.67%	39.67%	vanderLinden 2019
Volta	42.40%	3.00%	47.40%	16.00%	Bawah 2021
	31.40%	10.50%	10.0%	6.20%	Lokpo 2022
Western	18.75%	10.71%	18.75%		Osei-Yeboah 2018
Central	25.30%	19.00%	59.50%	7.60%	Acquah 2011
	8.69%	19.80%	14.90%	16.15%	Thomford 2023
	–	10.00%	–	–	Gato 2019
Greater Accra	43.75%	16.12%	28.95%	7.57%	Antwi-Baffour 2018
	30.00%	4.20%	67.50%	32.50%	Ofori 2018
	26.10%	21.70%	13.30%	67.20%	Tagoe 2019
	25.96%	19.20%	16.30%	35.60%	Tagoe 2020
	33.00%	11.25%	37.85%	45.90%	Ateko 2025

Overall, the distribution demonstrated considerable geographical variation in the burden and patterns of dyslipidaemia across Ghana, with a higher prevalence generally observed in the Southern and Middle Belts than in the Northern regions. However, the wide variability in reported lipid parameters across studies makes it challenging to derive a precise national estimate of the prevalence of dyslipidaemia.

## Discussion

This systematic review sought to determine the available evidence on the burden and patterns of dyslipidaemia among adult Ghanaians. The findings revealed wide variation in reported dyslipidaemia prevalence across studies, ranging from 3.0% to 72.4%, reflecting differences in study design, diagnostic criteria, population characteristics, and geographic coverage. Low HDL-C consistently emerged as the most common lipid abnormality, followed by elevated total cholesterol and LDL-C, whereas hypertriglyceridaemia was the least frequent. Overall, the available evidence indicates a substantial burden of dyslipidaemia in Ghana, particularly in urban and clinical populations, consistent with the broader epidemiological transition occurring in the country.

The prevalence patterns observed in this review broadly align with the regional trends reported across sub-Saharan Africa. In a meta-analysis by Noubiap et al. (2018), the pooled prevalence of low HDL-C was 37%, elevated total cholesterol was 25%, and elevated LDL-C was 28.6%, indicating that low HDL-C remains the dominant lipid abnormality across the continent [6]. However, this also raises the question of whether a comprehensive study should be conducted to categorise the SSA ranges. More importantly, this finding reveals a population-level disparity in lipid metabolism and environmental risk profiles that differs from Western trends, in which elevated TC is the most prevalent form of dyslipidaemia in Europe (54%) and the United States (48%) [12]. The implications for health outcomes are significant in this regard. HDL-C plays a crucial role in transporting cholesterol away from the arterial walls, thereby limiting the progression of atherosclerosis through anti-inflammatory and antioxidant pathways. The observed high prevalence of low HDL-C levels supports previous studies that reported a high burden and prevalence of cardiovascular diseases in the Ghanaian population [8,13]. Similarly, the significantly higher systolic and diastolic blood pressures in the diabetes and hypertension groups relative to the controls underscores the shared pathophysiology that links metabolic dysregulation, dyslipidaemia, and hypertension. The dominant prevalence of low HDL-C over elevated LDL-C levels also highlights the importance of validating Western-developed therapies in non-Western populations. In particular, statins, which are used to reduce cardiovascular risk and lower LDL-C levels, have limited effects on HDL-C levels [14]. Therefore, cardiovascular prevention strategies targeting HDL-C levels, such as lifestyle modifications, may require a greater emphasis in the Ghanaian context.

Studies that explored dyslipidaemia in people living with HIV/AIDS found elevated levels of VLDL-C relative to controls; however, this difference did not reach statistical significance. The impact of the virus on patients’ immunity and ongoing antiretroviral therapy may have introduced uncontrolled variables, contributing to the lack of statistical significance. Notably, Ghana shifted away from older Non-Nucleoside Reverse Transcriptase Inhibitor-based treatments towards Dolutegravir-based regimens as a first-line regimen in 2019 [15], whereas many of the studies sampled in this review were published well before that year. Additionally, the included studies reported specific antiretroviral regimens, treatment duration, and viral suppression status with varying levels of detail. Therefore, given that earlier Nucleoside Reverse Transcriptase Inhibitors (NRTIs), Non-Nucleoside Reverse Transcriptase Inhibitors (NNRTIs), and Protease Inhibitors (PIs) are known causes of altered lipid metabolism leading to dyslipidaemia [16], variation in antiretroviral treatment may have contributed to the observed lack of significance. Another complication in this patient population is that HIV infection, as a result of chronic inflammation, independently affects lipid metabolism and leads to dyslipidaemia [17]. Unfortunately, the included studies did not clearly distinguish between HIV-related and antiretroviral-related dyslipidaemia.

The current study also found a stark regional disparity in dyslipidaemia prevalence between the Northern Regions of Ghana and the Southern and Middle Belts. This observation parallels Ghana’s economic development and urbanisation trends. Indeed, studies in LMICs have identified a two-fold risk of developing cardiometabolic conditions as a result of residing in urban regions [18,19], which is associated with physical inactivity, smoking and urban dietary patterns. However, this observation must be interpreted in light of significant sampling bias, as 67% of participants were recruited from the urban south, namely the Ashanti, Greater Accra, and Central Regions of the country. The apparent regional disparities in dyslipidaemia prevalence may therefore point to differences in research infrastructure, access to healthcare, and health-seeking behaviour rather than population-level differences in disease burden. Another limitation arises from the overrepresentation of clinical populations compared with participants from community settings. Among the 24 included studies, 67% of the study population was hospital-based, and 33% were recruited from community settings. This likely inflated the prevalence estimates by oversampling individuals already seeking healthcare for metabolic, cardiovascular, and other chronic disorders. Without data on the true population-level burden in rural communities and the northern regions of Ghana, it is difficult to determine whether the results reflect genuine geographical variation in dyslipidaemia prevalence.

Study heterogeneity posed a challenge in synthesising data from the included studies. Differences in the diagnostic criteria used to define dyslipidaemia likely contributed to the wide prevalence range observed (4.0%–62.8%). Similar variability has been reported across sub-Saharan Africa, where studies employ different definitions, most commonly the NCEP ATP III criteria, alongside modified WHO thresholds or study-specific cut-offs. Across the region, dyslipidaemia prevalence varies substantially depending on the population studied, with community-based studies typically reporting prevalence around 20–25%, while much higher rates have been documented in high-risk groups such as individuals with hypertension (48–73%), HIV infection (~69%), or diabetes (up to 73–88%). These regional patterns are broadly consistent with the variability observed in the Ghanaian studies included in this review and likely reflect the ongoing epidemiological transition across many African countries, characterised by urbanisation, changing dietary patterns, and increasing cardiometabolic risk factors. The lack of standardised diagnostic criteria also complicates comparisons across studies and may limit the applicability of international guidelines developed in Western populations to African contexts with different baseline lipid profiles and cardiovascular risk patterns. Consequently, the heterogeneity of the available data precluded a formal meta-analysis.

Across the reviewed studies, sex-specific dyslipidaemia prevalence data were inconsistently reported, and the included studies recruited more females than males. This limits rigorous analysis of sex differences, which is of particular interest because sex differences in lipid profiles do exist. For instance, oestrogen is known to contribute to relatively favourable lipid profiles in premenopausal women, and these protective effects on lipid metabolism diminish after menopause [20]. Sex differences also exist in lifestyle risk factors for dyslipidaemia, such as dietary patterns, physical activity, alcohol consumption, and smoking. Taken together, the underreporting of sex-stratified data in the original studies limits the ability to clarify the true magnitude of sex differences in the dyslipidaemia burden.

In Ghana, dyslipidaemia is most often detected opportunistically during routine clinical care rather than through structured population-based screening programmes. Many adults are first diagnosed when seeking care for related conditions such as hypertension, diabetes, or other cardiometabolic disorders, or during workplace and community health screening events. Although Ghana’s National Health Insurance Scheme (NHIS) covers many essential health services, routine lipid screening for asymptomatic adults is not consistently implemented across healthcare settings. Consequently, dyslipidaemia frequently remains undiagnosed until individuals present with other cardiovascular risk factors or complications. The findings of the present review, therefore, highlight the need for improved surveillance and earlier detection strategies, including integrating lipid screening into existing non-communicable disease prevention programmes and into routine primary healthcare services. Such approaches align with the World Health Organisation’s recommendations for strengthening integrated cardiovascular risk screening and management within primary healthcare systems in low- and middle-income countries.

This systematic review shows that dyslipidaemia appears to be common among adult Ghanaians, with low HDL-C emerging as the dominant form across diverse study populations. However, precise national prevalence estimates are limited by the significant methodological heterogeneity and sampling bias. The evidence reviewed disproportionately represents urban clinical populations, whereas rural and non-clinical populations remain inadequately covered. Another limitation is the limited reporting of treatment and disease control in the included studies. Few studies provided information on whether dyslipidaemia, diabetes, or HIV infection were adequately controlled, and details regarding the class/ types of medication used were rarely reported. This lack of information limited the ability to evaluate treatment patterns. Nevertheless, the available data suggest that dyslipidaemia is common, particularly among Ghanaian adults, and given the established role of poor lipid metabolism in the development of cardiovascular disease, this warrants public health attention. This involves implementing nationally representative surveillance using standard, context-relevant diagnostic criteria for determining clinically significant dyslipidaemia, conducting research that addresses the identified knowledge gaps, and consolidating the lessons learned into comprehensive cardiovascular disease prevention strategies that are informed by locally relevant data rather than the uncritical adoption of approaches developed in other settings.

## Conclusion

This systematic review highlights that dyslipidaemia is highly prevalent among adult Ghanaians, with low HDL-C emerging as the most common lipid abnormality across diverse populations. The wide variation in prevalence across studies reflects methodological heterogeneity, differing diagnostic criteria, and predominance of data from urban and clinical populations. Nevertheless, overall evidence points to a growing cardiometabolic burden in Ghana, paralleling ongoing lifestyle and nutritional changes. Addressing this challenge will require harmonised diagnostic standards**,** population-based surveillance, and the integration of lipid screening into existing non-communicable disease prevention programmes. Future research should prioritise community-level studies that use context-appropriate lipid thresholds to improve the accuracy of national estimates and guide locally relevant cardiovascular risk-reduction strategies.

## Supporting information

S1 Appendix The study protocol. (PDF)

S1 Table Prisma checklist. (DOCX)

S2 Table Characteristics of the included studies. (DOCX)

S3 Table Quality and risk of bias assessment. (XLSX)

S1 Data Supplementary Data 1. (XLSX)

## References

[1] Berberich AJ, Hegele RA. A modern approach to dyslipidemia. Endocr Rev. 2022;43(4):611–53. doi: 10.1210/endrev/bnab037 34676866 PMC9277652

[2] Yusuf S, Joseph P, Rangarajan S, Islam S, Mente A, Hystad P, et al. Modifiable risk factors, cardiovascular disease, and mortality in 155 722 individuals from 21 high-income, middle-income, and low-income countries (PURE): A prospective cohort study. Lancet. 2020;395(10226):795–808. doi: 10.1016/S0140-6736(19)32008-2 31492503 PMC8006904

[3] Mocumbi AO. Cardiovascular health care in low- and middle-income countries. Circulation. 2024;149(8):557–9. doi: 10.1161/CIRCULATIONAHA.123.065717 38377254

[4] Obsa MS, Ataro G, Awoke N, Jemal B, Tilahun T, Ayalew N, et al. Determinants of dyslipidemia in Africa: A systematic review and meta-analysis. Front Cardiovasc Med. 2022;8:778891. doi: 10.3389/fcvm.2021.778891 35284497 PMC8904727

[5] Enoh JE, Akah RT, Nkeh-Chungag B. Cardiometabolic risk factors among African university students: A systematic review. BioMed. 2024;5(1):1.

[6] Noubiap JJ, Bigna JJ, Nansseu JR, Nyaga UF, Balti EV, Echouffo-Tcheugui JB, et al. Prevalence of dyslipidaemia among adults in Africa: A systematic review and meta-analysis. Lancet Glob Health. 2018;6(9):e998–1007. doi: 10.1016/S2214-109X(18)30275-4 30103999

[7] Konkor I, Kuuire VZ. Epidemiologic transition and the double burden of disease in Ghana: What do we know at the neighborhood level?. PLoS One. 2023;18(2):e0281639. doi: 10.1371/journal.pone.0281639 36827236 PMC9956066

[8] Agyekum F, Folson AA, Abaidoo B, Appiah LT, Adu-Boakye Y, Ayetey H, et al. Behavioural and nutritional risk factors for cardiovascular diseases among the Ghanaian population- A cross-sectional study. BMC Public Health. 2024;24(1):194. doi: 10.1186/s12889-024-17709-5 38229036 PMC10790451

[9] Obirikorang C, Osakunor DNM, Anto EO, Amponsah SO, Adarkwa OK. Obesity and cardio-metabolic risk factors in an urban and rural population in the Ashanti Region-Ghana: A comparative cross-sectional study. PLoS One. 2015;10(6):e0129494. doi: 10.1371/journal.pone.0129494 26046349 PMC4457529

[10] Page MJ, McKenzie JE, Bossuyt PM, Boutron I, Hoffmann TC, Mulrow CD, et al. The PRISMA 2020 statement: An updated guideline for reporting systematic reviews. BMJ. 2021;:n71. doi: 10.1136/bmj.n71 PMC8005924 33782057

[11] Stang A. Critical evaluation of the Newcastle-Ottawa scale for the assessment of the quality of nonrandomized studies in meta-analyses. Eur J Epidemiol. 2010;25(9):603–5. doi: 10.1007/s10654-010-9491-z 20652370

[12] Asadi Z, Moghbeli M, Khayyatzadeh SS, Mohammadi Bajgiran M, Ghaffarian Zirak R, Zare-Feyzabadi R, et al. A Positive Association between a Western Dietary Pattern and High LDL-C among Iranian Population. J Res Health Sci. 2020;20(3):e00485. doi: 10.34172/jrhs.2020.19 33169717 PMC7585768

[13] Doku A, Tuglo LS, Boima V, Agyekum F, Aovare P, Ali Abdulai M, et al. Prevalence of cardiovascular disease and risk factors in Ghana: A systematic review and meta-analysis. Glob Heart. 2024;19(1):21. doi: 10.5334/gh.1307 38404614 PMC10885824

[14] McTaggart F, Jones P. Effects of statins on high-density lipoproteins: a potential contribution to cardiovascular benefit. Cardiovasc Drugs Ther. 2008;22(4):321–38. doi: 10.1007/s10557-008-6113-z 18553127 PMC2493531

[15] Appiedu-Addo SNA, Appeaning M, Magomere E, Ansa GA, Bonney EY, Quashie PK. The urgent need for newer drugs in routine HIV treatment in Africa: the case of Ghana. Front epidemiol. 2025;5. 10.3389/fepid.2025.1523109 PMC11949944 40161547

[16] Calza L, Colangeli V, Manfredi R, Bon I, Re MC, Viale P. Clinical management of dyslipidaemia associated with combination antiretroviral therapy in HIV-infected patients. J Antimicrob Chemother. 2016;71(6):1451–65. doi: 10.1093/jac/dkv494 26846208

[17] Oka F, Naito T, Oike M, Imai R, Saita M, Inui A, et al. Correlation between HIV disease and lipid metabolism in antiretroviral-naïve HIV-infected patients in Japan. J Infect Chemother. 2012;18(1):17–21. doi: 10.1007/s10156-011-0275-5 21735099 PMC3278606

[18] Issaka A, Stevenson C, Paradies Y, Houehanou YCN, Bosu WK, Kiwallo JB, et al. Association between urban-rural location and prevalence of type 2 diabetes and impaired fasting glucose in West Africa: A cross-sectional population-based epidemiological study. BMJ Open. 2023;13(9):e063318. doi: 10.1136/bmjopen-2022-063318 37734888 PMC10514614

[19] Nsabimana P, Sombié OO, Pauwels NS, Boynito WG, Tariku EZ, Vasanthakaalam H, et al. Association between urbanization and metabolic syndrome in low- and middle-income countries: A systematic review and meta-analysis. Nutr Metab Cardiovasc Dis. 2024;34(2):235–50. doi: 10.1016/j.numecd.2023.07.040 38182494

[20] Nie G, Yang X, Wang Y, Liang W, Li X, Luo Q, et al. The effects of menopause hormone therapy on lipid profile in postmenopausal women: A systematic review and meta-analysis. Front Pharmacol. 2022;13:850815. doi: 10.3389/fphar.2022.850815 35496275 PMC9039020

