# Supplementary material for: Burden and patterns of dyslipidaemia among adult Ghanaians: A systematic review
Source: PLoS One. 2026 May 28;21(5):e0350185. doi: 10.1371/journal.pone.0350185 (PMC13218538; doi:10.1371/journal.pone.0350185)
Supplement: S1 Appendix — (PDF) [file pone.0350185.s001.pdf]

NIHR | National Institute for Health and Care Research

PROSPERO
International prospective register of systematic reviews

Prevalence of Dyslipidemia and Frequently Reported Dyslipidemias in Adult Ghanaians - A Systematic Review.

Richmond Ateko, Afua Adjei, Andrew Decker, Samuel Adadey, Eric Nyarko, Nicholas Thomford

To enable PROSPERO to focus on COVID-19 submissions, this registration record has undergone basic automated checks for eligibility and is published exactly as submitted. PROSPERO has never provided peer review, and usual checking by the PROSPERO team does not endorse content. Therefore, automatically published records should be treated as any other PROSPERO registration. Further detail is provided here.

Citation 1 change
Richmond Ateko, Afua Adjei, Andrew Decker, Samuel Adadey, Eric Nyarko, Nicholas Thomford. Prevalence of Dyslipidemia and Frequently Reported Dyslipidemias in Adult Ghanaians - A Systematic Review.. PROSPERO 2020 CRD42020198175. Available from https://www.crd.york.ac.uk/PROSPERO/view/CRD42020198175.

REVIEW TITLE AND BASIC DETAILS

Review title

Prevalence of Dyslipidemia and Frequently Reported Dyslipidemias in Adult Ghanaians - A Systematic Review.

Review objectives

1. To estimate the pooled prevalence of dyslipidaemia among adult populations in Ghana.

2. To assess the variation in dyslipidaemia prevalence across subgroups, including age, sex, and geographic setting (urban vs. rural).

3. To examine the types and patterns of dyslipidaemia (e.g., hypercholesterolemia, hypertriglyceridemia, low HDL-C, high LDL-C) reported in the Ghanaian adult population.

4. To evaluate the methodological quality of studies reporting dyslipidaemia prevalence in Ghana.

5. To identify gaps in the literature and inform future epidemiological surveillance and policy planning.

Review Questions

1. What is the overall prevalence of dyslipidaemia among adults in Ghana?

2. How does the prevalence of dyslipidaemia vary by sex, age group, or geographic location (e.g., urban vs. rural)?

3. What are the most common forms or patterns of dyslipidaemia reported in Ghanaian adults?

4. What diagnostic criteria and lipid thresholds have been used in studies conducted in Ghana?

5. What is the methodological quality of the included studies reporting on dyslipidaemia prevalence?

Keywords

Prevalence; Dyslipidemia; Ghana

SEARCHING AND SCREENING

Searches

We will search the following electronic databases: PubMed/MEDLINE, Embase, Scopus, Web of Science, African Journals Online (AJOL), and African Index Medicus (AIM). Grey literature will also be explored through Google Scholar and the University of Ghana Institutional Repository (UGSpace).

The search will include studies published from January 1, 1980, to February 28, 2025.

Restrictions:

Only studies published in English will be included. The review will be limited to studies involving human participants aged 18 years and older conducted within Ghana.

Study design

Only nonrandomized study types will be included.

Included
This review will include observational studies reporting the prevalence of dyslipidaemia among Ghanaian adults. Eligible study designs will include cross-sectional, cohort, and case-control studies. Studies must report quantitative estimates of dyslipidaemia prevalence or sufficient data to calculate it.

Excluded
Studies involving children and adolescents as well as studies on Ghanaians living abroad will be excluded. Case reports, case series, reviews, editorials, letters, and interventional trials will also be excluded.

Link to search strategy

A full search strategy has been uploaded to PROSPERO. The PDF may be accessed through this link
https://www.crd.york.ac.uk/PROSPEROFILES/70d79a8ae3ea7179ec61ea52d54941b0.pdf.

ELIGIBILITY CRITERIA

Condition or domain being studied

Dyslipidemia

Dyslipidemia is a disorder of rising global concern. It is a product of abnormal lipid metabolism, characterised by elevated levels of triglycerides (TG), total cholesterol (TC) and low-density lipoproteins (LDL) as well as reduced levels of high-density lipoproteins (HDL). These may manifest individually or in combination. Dyslipidemia is positively associated with obesity, atherosclerotic risk, type 2 diabetes mellitus, metabolic syndrome, and premature cardiovascular disease. According to the National Cholesterol Education Program/Adult Treatment Panel III (NCEP/ATP III), dyslipidemia is defined as TC > 5.17 mmol/L, TG > 1.7 mmol/L, LDL-C > 3.36 mmol/L, and HDL-C < 1.03 mmol/L for males and < 1.3 mmol/L for females. The European Atherosclerosis Society defines dyslipidemia as TC > 5.2 mmol/L, TG > 1.75 mmol/L, LDL-C > 3.5 mmol/L, HDL-C < 0.9 mmol/L, and atherogenic index > 5.8. The World Health Organization (WHO) defines it as TC > 5.2 mmol/L, TG 1.7 mmol/L, LDL-C > 3.5 mmol/L and HDL-C < 0.9 mmol/L in males and 1.0 mmol/L in females.

Population

Included
Adult Ghanaians older than 18 years (apparently healthy controls or patients diagnosed with T2DM, HIV, or hypertension)

Excluded
Children and adolescents under 18 years of age.

Intervention(s) or exposure(s)

Included
The objective of this review is not to assess interventions for dyslipidemias but rather to highlight the prevalence of elevated total cholesterol, triglycerides, low-density lipoproteins and reduced serum high-density lipoproteins according to all three standardised definitions of dyslipidemia (NCEP/ATP III, WHO and European Atherosclerosis Society definitions)

Comparator(s) or control(s)

This review does not have any comparators

Context

Studies conducted in diabetes outpatient clinics, hospitals, community settings (such as churches and mosques), and residential neighbourhoods in urban and rural areas, as well as institutional and workplace environments, will be included.

OUTCOMES TO BE ANALYSED

Main outcomes

The primary outcome of interest is the prevalence of dyslipidaemia among adult populations in Ghana.

Outcome definition:

Dyslipidaemia will be defined according to standard clinical thresholds, including but not limited to:

• Total cholesterol (TC) ≥ 5.2 mmol/L

• Low-density lipoprotein cholesterol (LDL-C) ≥ 3.4 mmol/L

• High-density lipoprotein cholesterol (HDL-C) less than 1.0 mmol/L for men or less than 1.3 mmol/L for women

• Triglycerides (TG) ≥ 1.7 mmol/L

Studies using national, WHO, NCEP ATP III, or other internationally recognised criteria will be included, and diagnostic thresholds will be recorded for comparability.

Measurement instruments:

Acceptable measurements include:

• Venous blood lipid profiles assessed via enzymatic colorimetric methods

• Point-of-care testing devices, if validated and documented

Time points:

Only baseline or cross-sectional time points will be considered. For cohort studies, only baseline prevalence data will be extracted.

Measures of effect
The primary effect measure will be the prevalence (%) of overall dyslipidaemia, as well as specific subtypes (e.g., hypercholesterolemia, low HDL-C). These will be pooled using random-effects meta-analysis to account for heterogeneity across studies.

Additional outcomes
Where data are available, secondary outcomes will include:

Prevalence of individual lipid abnormalities, including:

- Hypercholesterolemia
- Hypertriglyceridemia
- Low HDL-C
- Elevated LDL-C

Prevalence stratified by:

- Sex (male vs. female)
- Age groups (e.g., 18–39, 40–59, ≥60 years)
- Geographic location (urban vs. rural settings)
- Study setting (e.g., hospital-based vs. community-based

Measures of effect
Same as the main outcome.

DATA COLLECTION PROCESS

Data extraction (selection and coding)

Two investigators will independently conduct a blinded screening of journals based on inclusion and exclusion criteria, download citations into EndNote referencing software and remove duplicates. Screening results will then be compared and merged. Extracted data will include 1) Last name of first author2) Date of publication3) Serum levels of triglycerides, total cholesterol, low density lipoproteins and high density lipoproteins 4) Types of dyslipidemia5) Sample size6) Age range, mean and median age7) Study location8) Sampling period9) Number of cases of dyslipidemiaExtracted data will be manually input into an Excel spreadsheet and will be analyzed using SPSS version 25 and R. Should any disagreement arise during the screening and data extraction process, a third party who is an expert in the field will be consulted.

Risk of bias (quality) assessment

The following study characteristics will be extracted and assessed:

• Study design (e.g., cross-sectional, cohort)

• Study setting (e.g., hospital-based, community-based, institutional)

• Geographic location (urban vs. rural; region within Ghana)

• Sample size

• Sampling method (e.g., random, convenience)

• Participant characteristics (e.g., age range, sex distribution)

• Diagnostic criteria used for dyslipidaemia

• Measurement methods (e.g., laboratory-based tests, point-of-care devices)

• Year(s) of data collection and publication year

Risk of bias/quality assessment:

The Joanna Briggs Institute (JBI) Critical Appraisal Checklist for Studies Reporting Prevalence data will be used to assess the methodological quality and risk of bias of included studies. This tool evaluates key domains such as:

• Sample representativeness

• Sampling technique

• Adequacy of sample size

• Reliability of outcome measurement

• Use of appropriate statistical analysis

Two reviewers will independently assess the risk of bias. Disagreements will be resolved through discussion or consultation with a third reviewer.

PLANNED DATA SYNTHESIS

Strategy for data synthesis

Quantitative data synthesis will be conducted where sufficient comparable data are available.

Descriptive Synthesis:

A narrative synthesis will first be presented, summarizing:

• Study characteristics (design, setting, population)

• Prevalence estimates of overall dyslipidaemia and its subtypes

• Diagnostic criteria and measurement methods used

• Variations across regions, population groups, and study settings

Meta-analysis:

If ≥2 studies provide prevalence data with sufficient methodological similarity, a meta-analysis will be conducted using random-effects models to account for between-study variability.

• Effect measure: The primary summary measure will be the pooled prevalence (%) with 95% confidence intervals (CI).

• Prevalence proportions will be transformed using the double arcsine transformation to stabilize variance and back-transformed for interpretation.

• Heterogeneity will be assessed using the I^2 statistic and Cochran's Q test.

Publication Bias:

If ≥10 studies are included, funnel plots will be generated, and Egger's test will be used to assess potential publication bias.

All statistical analyses will be conducted using Stata or R.

Analysis of subgroups or subsets

Subgroup and Sensitivity Analyses:

Where possible, subgroup analyses will explore prevalence differences by:

• Sex (male vs. female)

• Age groups

• Geographic setting (urban vs. rural)

• Study setting (hospital, community, workplace)

Sensitivity analyses will be performed by excluding studies at high risk of bias to evaluate the robustness of pooled estimates.

REVIEW AFFILIATION, FUNDING AND PEER REVIEW

Review team members 1 change

Dr Richmond Ateko (review guarantor). University of Ghana. Ghana.

No conflict of interest declared.

Afua Adjei. University of Ghana. Ghana.

No conflict of interest declared.

Andrew Decker. University of Ghana. Ghana.

No conflict of interest declared.

Dr Samuel Adadey. University of Cape Town. Ghana.

No conflict of interest declared.

Dr Eric Nyarko. University of Ghana. Ghana.

No conflict of interest declared.

Dr Nicholas Thomford. University of Cape Coast. Ghana.

No conflict of interest declared.

Review affiliation

University of Ghana

Funding source

Review has no funding and no agreed support from an academic institution and is done in authors' own time.

TIMELINE OF THE REVIEW

Review timeline 1 change

Start date: 1 August 2020. End date: 30 November 2025.

Date of first submission to PROSPERO

21 October 2020

Date of registration in PROSPERO

21 November 2020

CURRENT REVIEW STAGE

Publication of review results

The intention is to publish the review once completed.The review will be published in English

Stage of the review at this submission

Review stage	Started	Completed
Pilot work	✓	✓
Formal searching/study identification	✓	✓
Screening search results against inclusion criteria	✓	✓
Data extraction or receipt of IPD	✓	✓
Risk of bias/quality assessment	✓	✓
Data synthesis	✓	✓

Review status

The review is completed.

ADDITIONAL INFORMATION

Review conflict of interest
None known

Country
Ghana

Medical Subject Headings
Adult; Dyslipidemias; Ghana; Humans; Prevalence

Revision note 1 change
Changes were made because of updates to the review team's composition and author roles. These adjustments ensure that the record reflects the correct contributors and their current roles in the review.

Disclaimer
The content of this record displays the information provided by the review team. PROSPERO does not peer review registration records or endorse their content.

PROSPERO accepts and posts the information provided in good faith; responsibility for record content rests with the review team. The guarantor for this record has affirmed that the information provided is truthful and that they understand that deliberate provision of inaccurate information may be construed as scientific misconduct.

PROSPERO does not accept any liability for the content provided in this record or for its use. Readers use the information provided in this record at their own risk.

Any enquiries about the record should be referred to the named review contact
