# Supplementary material for: Burden and patterns of dyslipidaemia among adult Ghanaians: A systematic review
Source: PLoS One. 2026 May 28;21(5):e0350185. doi: 10.1371/journal.pone.0350185 (PMC13218538; doi:10.1371/journal.pone.0350185)
Supplement: S2 Table — (DOCX) [file pone.0350185.s003.docx]

**Table 2. Characteristics of included studies**

| **Author**  **(publication year)** | **Region** | **Study area** | **Study setting** | **Healthy/**  **diseased** | **Sample size**  **(n)** | **Men (n)** | **Age range** | **Diagnostic cut-off used** | **Lipid parameter**  **Reported** | **Dyslipidaemia**  **Prevalence***  **(%)** |
| --- | --- | --- | --- | --- | --- | --- | --- | --- | --- | --- |
| Acquah (2011) | Central | Urban | Hospital | Diabetics | 79 | 22 | 27-90 | NCEP ATP III | TC, TG, LDL-C, HDL-C | 7.6 – 55.7 |
| Agogo  (2018) | Northern | Rural | Community | Healthy | 1839 | 846 | 40-60 | ESC | TC, TG, LDL-C, HDL-C | NS |
| Alidu  (2023) | NS | Urban | Hospital | Diabetics | 121 | 121 | NS | ND | TC, TG, LDL-C, HDL-C | 63.8 |
| Anto  (2019) | Bono | Urban | Hospital | Diabetics | 215 | 86 | 20-79 | ADA | TC, TG, LDL-C, HDL-C | 47.4 - 66.5 |
| Antwi-Baffour  (2018) | Greater. Accra | Urban | Hospital | Diabetics | 304 | 171 | 28-70 | KBTH | TC, TG, LDL-C, HDL-C | NS |
| Asamoah-Boakye  (2017) | Ashanti | Urban | Hospital | Diabetics | 152 | 37 | 35-80 | NCEP ATP III | TC, TG, LDL-C, HDL-C | 63.8 |
| Ateko  (2025) | Greater. Accra | Urban | Community and hospital | Healthy and Diabetics | 970 | 469 | 20-88 | NCEP ATP III | TC, TG, LDL-C, HDL-C |  |
| Bani  (2020) | Northern | Urban | Hospital | HIV/AIDS | 118 | 19 | 19-71 | NCEP ATP III | TC, TG, LDL-C, HDL-C | 39.0 |
| Bawah  (2021) | Volta | Urban | Hospital | Diabetics | 125 | 36 | 36-86 | NCEP ATP III | TC, TG, LDL-C, HDL-C | 3.0 - 42.0 |
| Eghan  (2003) | Ashanti | Urban | Hospital | Healthy, diabetics, hypertensives | 248 | 103 | 22-85 | NCEP ATP III | TC, TG, LDL-C, HDL-C | 25 - 72.4 |
| Gato  (2019) | Central | Urban and rural | Community | Healthy | 70 | 42 | NS | NCEP ATP III | TC, TG, LDL-C, HDL-C | NS |
| Kodaman  (2016) | Bono | Urban and rural | Community | Healthy | 3317 | 1441 | 18-99 | NCEP ATP III | TC, TG, LDL-C, HDL-C | NS |
| Li  (2020) | Greater Accra, Ashanti, Central, Northern | Urban and rural | Community | Healthy, diabetics, hypertensives | 1106 | 464 | ≥ 18 | NCEP ATP III | TC, TG, LDL-C, HDL-C | 40.8 |
| Lokpo  (2022) | Volta | Urban | Hospital | Diabetics | 210 | 96 | 20-70 | NCEP ATP III | TC, TG, LDL-C, HDL-C | 67.1 |
| Micah  (2012) | Ashanti | Urban | Hospital | Healthy, diabetics, hypertensives | 424 | 163 | 20-88 | ESC | TC, TG, LDL-C, HDL-C | NS |
| Ngala  (2013) | Ashanti | Urban | Hospital | HIV-AIDS | 305 | 97 | 20-64 | NCEP ATP III | TC, TG, LDL-C, HDL-C | NS |
| Obirikorang  (2015) | Ashanti | Urban and rural | Community | Healthy | 672 | 312 | 20-79 | NCEP ATP III | TC, TG, LDL-C, HDL-C | Varying |
| Obirikorang  (2020) | NS | NS | NS | HIV-AIDS | 289 | 59 | NS | NCEP ATP III | TC, TG, LDL-C, HDL-C | 31.1 |
| Ofori  (2018) | GT. Accra | Urban | Community | Healthy | 120 | 20 | NS | NCEP ATP III | TC, TG, LDL-C, HDL-C | NS |
| Osei-Yeboah  (2018) | Western | Rural | Hospital | Healthy | 112 | 48 | 22-59 | ND | TC, TG, | 26.8 |
| Tagoe  (2019) | GT. Accra | Urban | Hospital | HIV-AIDS | 180 | 65 | NS | NCEP ATP III | TC, TG, LDL-C, HDL-C | NS |
| Tagoe  (2020) | GT. Accra | Urban | Hospital | Healthy,  Breast cancer | 104 | 0 | NS | NCEP ATP III | TC, TG, LDL-C, HDL-C | NS |
| Thomford  (2023) | Central | Urban and rural | Hospital | HIV-AIDS; Malaria; | 161 | 46 | 19-79 | NCEP ATP III | TC, TG, LDL-C, HDL-C | Varying |
| Vander Linden  (2019) | Ashanti | Urban and rural | Community | Healthy | 2468 | 813 | 27-70 | ESC | TC, TG, LDL-C, HDL-C | Varying |

NS- Not stated; ND- Not defined; ATP III- Adult Treatment Panel III; ESC- European Society of Cardiology; ADA- American Diabetes Association; KBTH- Korle-Bu Teaching Hospital; TC- Total cholesterol; TG- Triglyceride; LDL-C- Low-density lipoprotein cholesterol; HDL-C- High-density lipoprotein cholesterol. * refers to the overall or range of prevalence of dyslipidaemia in the study population.
